# Successful Remove of a Metal Axletree Causing Penile Strangulation in a 19-Year-Old Male by Degloving Operation

**DOI:** 10.1155/2012/532358

**Published:** 2012-08-09

**Authors:** Weidong Gan, Rong Yang, Changwei Ji, Huibo Lian, Hongqian Guo

**Affiliations:** Department of Urology, Nanjing Drum Tower Hospital' The Affliated Hospital of Nanjing University Medical School, Nanjing, Jiangsu 210008, China

## Abstract

Penile strangulation caused by foreign bodies mostly occurs in adolescents and adult males. When it happens, foreign bodies are often not easy to be removed. Penile strangulation is a rarely described urological emergency, especially in the adolescent population. This paper demonstrates the successful removal of a metal axletree causing penile strangulation in a 19-year-old male with the help of degloving operation.

## 1. Introduction

Penile strangulation is rare and usually results from placement of constricting objects to enhance sexual performance. Nonmetallic and thin objects are easy to remove but can cause severe injury. Metallic objects are difficult to remove while the injuries are usually less severe [1]. Penile ulceration and edema in children may well indicate the presence of strangulation objects. Nonmetallic and thin metallic objects can be removed more easily compared with heavy metallic objects. Cutting is the most common method described, although appropriate cutting tools may be difficult to obtain and the process may be tedious with the possibility of iatrogenic penile injury [2]. In this paper, we will present a new method to remove such objects by degloving operation.

## 2. Case Report

A 19-year-old male presented to the emergency room with a strangulating metal axletree on his penis down to the penoscrotal junction. He placed the axletree 14 days ago. He thought it would be easy to be removed after penile erection, but as time went by, his penis had suffered severe edema, thus preventing its removal. He was so ashamed that he did not go to see the doctor. Two weeks later there were ruptures of the penis skin near the axletree with unpleasant smell. With the symptom of dysuria, he had to come to hospital. It was admitted that he had three similar performances before, and it did not take his time to remove the axletree. He said there was special euphoria when he was doing this.

**Figure 1:**
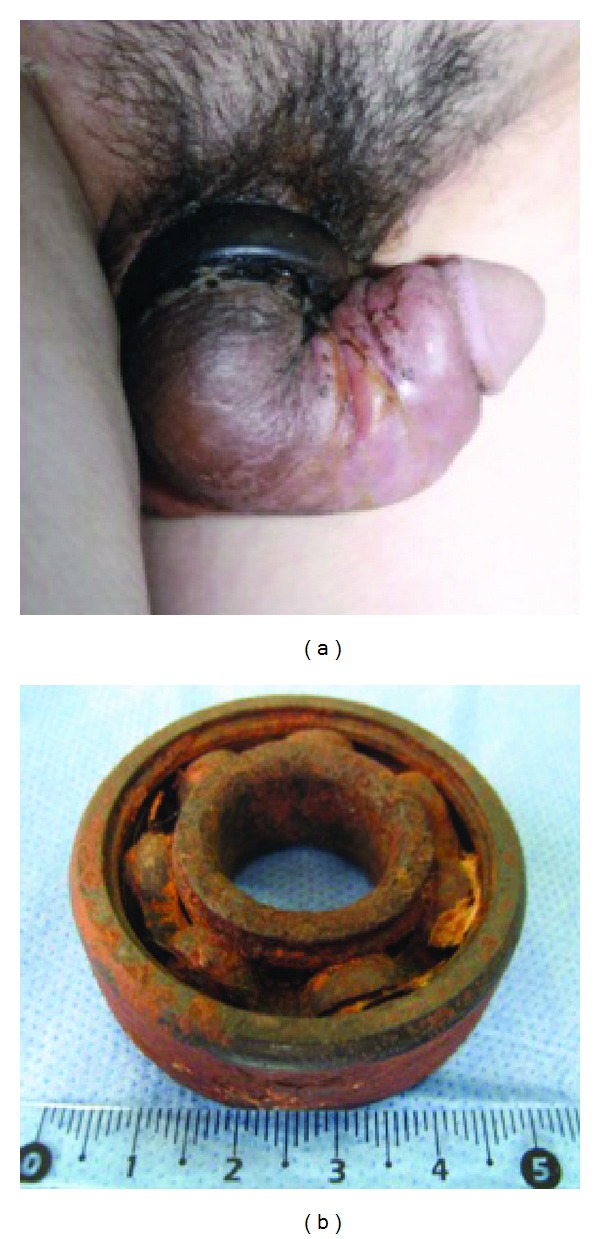
(a) Axletree strangulated to the penoscrotal junction; (b) removed axletree.

When he arrived, we saw the basilar part of the penis was incarcerated by the axletree, and it could still turn round (Figure 1(a)). The skin under the axletree was broken, and there were local necrosis. Distal part of the penis was obviously crooked and swelling, and its diameter was significantly greater than that of the axletree. We measured the axletree and its thickness and width were both 1.5 cm. So we gave up the intention of cutting it.

A 21G needle was punctured into the cavernosum to exsanguinate the blood, and the shrinkage of distal penis was expected. However, we found the mainly swelling part was the foreskin. Obviously, this method had no effect. At that time deglove operation was almost the only attempt left. Under epidural anesthesia, we circlely incised the foreskin at the distal part of the strangulated area, degloving the skin just above the albuginea till reaching the penis coronal part. After that, we found the diameter of the penis was approximately equal to the inner diameter of the axletree. With the help of lubricants, we slowly moved the axletree to the distal part of the penis in following sequence: the penis without skin, coronal part, and the degloved foreskin. The strangulated skin by the axletree was dark and lack of vitality, so we had to cut it circlely with a width of 0.5 cm. Then we sutured the distal degloved penile tissue flap and the proximal foreskin layers with 4–0 Vicryl. The penis was properly bandaged with some pressure. An 18 Fr Folley's catheter was placed. The weight of the axletree was 135.5 g, and the outer and inner diameters were 5.1 cm and 2.1 cm, respectively (Figure 1(b)).

Intravenous antibiotics (Cefuroxime) were used pre- and three days postoperation. No microcirculation regulator or local drugs were used. Twenty-four hours later the catheter was removed and the patient could urinate without difficulty. The edema of penile skin subsidised gradually. In 10, days the Vicryl was stitched and the skin flap was healed without any tissue loss. No voiding dysfunction occured in this patient in 2 year's followup. He had normal erection without insularity.

## 3. Discussion

Penile strangulation mostly occurs in adolescents and adult males under the desire of sexual curiosity. They often use foreign bodies to increase the penile rigidity and prolong the time of erection. Although they could acquire impermanence pleasure, the foreign bodies are often unable to be removed after congestive erection. Due to psychological shameness and lack of medical knowledge, they just tend to deal with them simply and crudely. Thus, foreign bodies usually cannot be removed and penis is often injured. Guilty, fearing of blame, and humiliation prevent them from going to the hospital when they failed to remove the foreign bodies. Generally only when there are serious complications, they would like to seek medical help. They could also be sent to hospital by their families, but the best time point has often been missed then.

There are many ways to remove the strangulated foreign bodies, such as aspiration method [3, 4], cutting method [5, 6], and deglove operation [7]. No matter what kinds of foreign bodies they were, we should deal with them from simple to complex, and try from less harmless ones. Lubricant is our first choice. We can also exsanguinate the blood of the penis as well as using the lubricants for the more complicate case. When we deal with plastic textures or soft metal materials which are not too hard, we can directly cut them and the penis should be properly protected at the same time. Dental drill, electric grinders, and other cutting instruments are commonly used. Cold running water was usually employed to prevent thermal damage, especially when we were cutting the part closing to the skin strangulated. At last, for those hard ones such as axletrees, steel, and other hard materials which cannot be easily cut, deglove operation should come to our mind.

Deglove operation is very useful when we want to remove the hard foreign bodies quickly. We first incise the foreskin of the penile at the distal margin of the strangulated area. We gradually dissociate the skin to coronal department. In order to minimize the size of the distal penis, we have to exsanguinate the blood of it. If necessary, we can also exsanguinate the blood from the glans. We must pay close attention to the blood supply of the foreskin during and after operation. Once we found there were signs of ischemia or necrosis, we should have no hesitate to remove the rotted skin, and then inosculate the proximal and distal skin flaps. In order to ensure the blood supply of flaps, we should dissociate the foreskin as close to albuginea as possible. It is best to dissociate appropriately the proximal flaps down to the penoscrotal junction first, if we want to reduce the tension of the flaps and avoid the insularity of penis after operation.
